# Skin Photodamage and Melanomagenesis: A Comprehensive Review

**DOI:** 10.3390/cancers17111784

**Published:** 2025-05-26

**Authors:** Michele Manganelli, Giorgio Stabile, Camila Scharf, Antonio Podo Brunetti, Giovanni Paolino, Roberta Giuffrida, Gianmarco Diego Bigotto, Giuseppe Damiano, Santo Raffaele Mercuri, Fabio Sallustio, Eleonora Mangano, Roberta Bordoni, Paola De Nardi, Gabriella Guida, Caterina Foti, Giuseppe Argenziano, Caterina Longo, Giovanni Pellacani, Nathalie Rizzo, Vincenzo Russo, Stefania Guida, Franco Rongioletti

**Affiliations:** 1 Department of Translational Biomedicine and Neuroscience, University of Bari Aldo Moro, 70121 Bari, Italy; m.manganelli1991@gmail.com (M.M.); gabriella.guida@uniba.it (G.G.); 2 Dermatology Clinic, IRCCS San Raffaele Hospital, 20132 Milan, Italy; stabile.giorgio@hsr.it (G.S.); podobrunetti.antonio@hsr.it (A.P.B.); bigotto.gianmarco@hsr.it (G.D.B.); guida.stefania@hsr.it (S.G.); rongioletti.franco@hsr.it (F.R.); 3 Faculty of Medicine, Vita-Salute San Raffaele University, 20132 Milan, Italy; paolino.giovanni@hsr.it (G.P.); mercuri.santoraffaele@hsr.it (S.R.M.); 4 Dermatology Unit, Department of Mental and Physical Health and Preventive Medicine, University of Campania Luigi Vanvitelli, 80138 Naples, Italy; giuseppe.argenziano@unicampania.it; 5 Dermatology and Cosmetology, San Raffaele Hospital, 20132 Milano, Italy; 6 Department of Clinical & Experimental Medicine, Section of Dermatology, University of Messina, 98122 Messina, Italy; giuffrida.roberta@unime.it; 7 Immuno-Biotherapy of Melanoma and Solid Tumors Unit, Division of Experimental Oncology, San Raffaele Scientific Institute, DIBIT, Via Olgettina 58, 20132 Milan, Italy; damiano.giuseppe@hsr.it (G.D.); russo.vincenzo@hsr.it (V.R.); 8 Department of Precision and Regenerative Medicine and Ionian Area, Aldo Moro University of Bari, 70121 Bari, Italy; fabio.sallustio@uniba.it; 9 Institute of Biomedical Technologies (ITB), National Research Council (CNR), Segrate, 20054 Milan, Italy; eleonora.mangano@itb.cnr.it (E.M.); roberta.bordoni@itb.cnr.it (R.B.); 10 Colorectal Surgery, IRCCS San Raffaele Scientific Institute, Via Olgettina 60, 20132 Milan, Italy; denardi.paola@hsr.it; 11 Section of Dermatology and Venereology, Department of Precision and Regenerative Medicine and Jonian Area, University of Bari “Aldo Moro”, 70124 Bari, Italy; caterina.foti@uniba.it; 12 Department of Dermatology, University of Modena and Reggio Emilia, 41121 Modena, Italy; caterina.longo@unimore.it; 13 Skin Cancer Center, Azienda Unità Sanitaria Locale, IRCCS di Reggio Emilia, 42122 Reggio Emilia, Italy; 14 Department of Dermatology, Sapienza University, 00185 Rome, Italy; pellacani.giovanni@uniroma1.it; 15 Department of Pathology, IRCCS San Raffaele Scientific Institute, 20132 Milan, Italy; rizzo.nathalie@hsr.it

**Keywords:** melanoma, melanomagenesis, skin photodamage, ultraviolet radiation, cumulative sun damage

## Abstract

Melanoma, the most aggressive form of skin cancer, still represents a significant and growing public health concern. Ultraviolet radiation (UVR) is considered the primary driver of melanoma, although genetic predisposition also plays a critical role. This review explores the intricate molecular mechanisms by which UVR-induced photodamage contributes to melanoma development. We examine epidemiological evidence linking UV exposure to increased risk, detailing how UVR damage to DNA triggers inflammatory responses and impairs DNA repair mechanisms. Specifically, we discuss the roles of nucleotide excision repair (NER) and base excision repair (BER) in mitigating UV damage. The review further explores diagnostic and surgical implications for melanomas arising on sun-exposed skin. By synthesizing current evidence, this overview aims to deepen understanding of the complex relationship between UVR, photodamage, and melanoma, shedding light on the need for personalized preventive strategies to better stratify the risk and introduce behavioral changes to reduce skin photodamage.

## Simple Summary

Melanoma is the most aggressive form of skin cancer, and its increasing incidence is a major public health concern. One of the main causes of melanoma is exposure to ultraviolet radiation from the sun, which damages the DNA in skin cells and increases the risk of cancer. However, genetics also play a role in determining who is more likely to develop this disease. This overview aims to explore how ultraviolet radiation damages skin cells at a molecular level, how the body tries to repair this damage, and why these repair processes sometimes fail, leading to melanoma. The study also examines different types of melanoma that develop in sun-exposed skin and how they are classified based on the amount of sun damage they have accumulated over time. By improving our understanding of these processes, this research could help develop better strategies for preventing melanoma and identifying individuals at higher risk, ultimately leading to earlier detection and improved treatment decisions.

## 1. Introduction

Melanoma is one of the most aggressive and rapidly increasing skin cancers, originating from melanocytes, the pigment-producing cells of the skin. While a subset of melanomas develops in sun-protected areas, and approximately 10% of cases have a genetic predisposition, ultraviolet radiation (UVR) remains the leading environmental factor in cutaneous melanoma (CM) development, accounting for 60–70% of cases [1].

UVR, including UVA and UVB radiation, is a well-established carcinogen that induces extensive skin cell damage and plays a pivotal role in melanomagenesis [1]. They have distinct but overlapping carcinogenic roles: while UVB directly causes DNA mutations, UVA contributes through oxidative stress and immune evasion. Recent studies have elucidated the molecular pathways by which UV-induced photodamage contributes to tumorigenesis. These processes involve both direct DNA damage and subsequent inflammatory responses, leading to a cascade of events that promote melanocyte transformation [2,3].

DNA mutations accumulate in key regulatory genes, disrupting cellular processes such as cell cycle regulation, apoptosis, and DNA repair [4]. In response to UV-induced damage, skin cells employ repair mechanisms such as nucleotide excision repair (NER) and base excision repair (BER) pathways [5,6,7,8,9,10,11,12]. However, chronic UV exposure can overwhelm these pathways, leading to persistent inflammation, microenvironmental changes, and immune evasion, all of which contribute to melanoma development [13]. Molecular mechanisms underlying melanoma progression highlighted the pivotal role of V600BRAF mutation in switching on metabolic reprogramming [14]. Furthermore, the induction of autophagy has been suggested to be a pro-survival mechanism for melanoma cells [15,16,17]. While an initial increase in autophagy due to UVR might be a cellular defense mechanism, a dysregulated or sustained high level of autophagic flux in melanoma cells could paradoxically contribute to their survival, growth, and spread [1].

Usually, melanoma progresses following a characteristic sequence from the radial growth phase (RGP) to the vertical growth phase (VGP). In the RGP, atypical melanocytes proliferate laterally within the epidermis or superficial dermis, remaining in situ and non-invasive. The transition to the VGP marks a critical event, where melanoma cells gain the ability to invade deeper dermal layers and potentially metastasize. This shift is often driven by cumulative UVR-induced mutations and microenvironmental changes. Understanding the molecular signals regulating this progression is essential for early diagnosis and therapeutic intervention [18].

This review will explore the mechanisms linking UV-induced photodamage to melanoma, focusing on key epidemiological, molecular, and pathophysiological aspects. Additionally, we will examine diagnostic challenges, surgical considerations, and controversies regarding the role of chronic sun exposure in melanoma pathogenesis. By synthesizing epidemiological trends, DNA repair mechanisms, inflammation, and immunological consequences of UVR exposure, this review provides an integrated framework connecting photodamage to melanomagenesis. Particular emphasis is placed on the translational relevance of molecular alterations, including their implications for diagnosis, biomarker development, and therapeutic responsiveness. This comprehensive approach aims to support a deeper understanding of the complex interplay between UVR, skin aging, and melanoma, ultimately informing both preventive strategies and personalized clinical management.

## 2. Epidemiology

The incidence of CM has risen dramatically in recent decades, posing an increasing public health concern. CM is most commonly diagnosed between the ages of 50 and 60, with a mean age of 59 years [19]. Gender differences are evident, with men more frequently developing melanoma on the trunk and upper limbs, whereas women are more prone to melanomas on the lower extremities [20]. Skin type is also a key risk factor, with Fitzpatrick type I individuals—who are more susceptible to photodamage—at the highest risk [21].

Large-scale epidemiological studies, such as GLOBOCAN [22] and the Nurses’ Health Study [23], have demonstrated a strong correlation between sun exposure and melanoma incidence. According to GLOBOCAN 2020, melanoma accounts for over 324,000 new cases and 57,000 deaths globally each year. European incidence rates vary widely, with the highest observed in northern countries such as Norway and Sweden, and comparatively lower—but steadily increasing—rates in southern countries. In Italy, data from the AIRTUM registry (Associazione Italiana Registri Tumori) estimate an incidence of approximately 12–15 new melanoma cases per 100,000 inhabitants annually, with an upward trend particularly among men and older individuals [24]. These findings highlight the need for region-specific strategies to support targeted prevention campaigns and early detection, particularly in aging populations with high cumulative UVR exposure.

Chronic UV exposure, particularly in populations with high cumulative exposure, is associated with a significant increase in melanoma risk, especially in situ and head and neck melanomas, mainly those classified as lentigo maligna/lentigo maligna melanoma (LM/LMM) [25]. However, some studies suggest a paradoxical protective effect of continuous occupational sun exposure, (OR = 0.57) [26] possibly due to increased melanin production and epidermal thickness, through adaptive photoprotection mechanisms.

Instead, intermittent sun exposure, particularly episodes of intense exposure leading to sunburns, has been identified as a stronger risk factor than chronic exposure. Studies have reported an odds ratio (OR) of 3.00 for individuals with frequent sunbathing habits and 3.90 for those with a history of sunburns [26]. These findings highlight the complex interplay between different patterns of sun exposure and melanoma risk and its understanding is crucial to refining preventive strategies and improving early diagnosis.

## 3. UVR-Induced DNA Damage

UVR is the most significant environmental factor driving melanoma development, with its ability to induce DNA damage and disrupt cellular homeostasis in the skin. The UVR spectrum comprises three main wavebands: UVA (315–400 nm), UVB (280–315 nm), and UVC (200–280 nm). While UVC is completely absorbed by the stratospheric ozone layer (O_3_) and thus biologically irrelevant, both UVA and UVB reach the Earth’s surface and contribute to skin carcinogenesis [27].

The effects of UVR on DNA are well-documented, yet the distinct pathways through which UVA and UVB contribute to melanomagenesis remain a topic of ongoing research. UVB, with its higher energy, directly affects DNA by inducing cyclobutane pyrimidine dimers (CPDs) and 6-4 photoproducts (6-4PPs), leading to helix distortions that impair normal replication and transcription [28,29,30,31]. If left unrepaired, these lesions accumulate and generate UVB signature mutations, specifically C>T and CC>TT transitions, frequently observed in key tumor suppressor genes such as *TP53* and *CDKN2A* [32,33,34,35].

On the other hand, UVA radiation, though less energetic, penetrates deeper into the dermis, and exerts its carcinogenic effects through reactive oxygen species (ROS) generation. The increase in superoxide radical ions (O_2_^−^) upon UVA exposure sets off a cascade of oxidative stress, leading to secondary DNA damage [36]. The enzymatic activity of NADPH oxidase (NOX) further contributes to this oxidative burst, while inducible nitric oxide synthase (iNOS) generates nitric oxide (NO), which reacts with superoxide to produce peroxynitrite (ONOO^−^), a highly reactive molecule that diffuses into the cell and induces damage [37]. The interaction between ONOO^−^ and melanin monomers leads to the formation of dioxetane intermediates, which undergo spontaneous thermolysis, producing highly energetic carbonyl compounds. These compounds, in turn, transfer UV energy directly to DNA, leading to additional CPD formation [38].

The role of melanin in this process is particularly intriguing. While eumelanin, the black-brown pigment, provides a degree of photoprotection by absorbing and dissipating UV energy, pheomelanin, the red-yellow pigment, contributes to ROS generation, paradoxically enhancing UVR-induced DNA damage [39]. Studies have demonstrated that melanocytes rich in pheomelanin accumulate twice as many CPDs as their eumelanin-producing counterparts, a phenomenon particularly relevant in individuals with red hair. These individuals frequently carry *MC1R* polymorphisms, which not only reduce eumelanin production but also impair DNA repair mechanisms, further amplifying melanoma risk [40]. Beyond its photoprotective limitations, pheomelanin also participates in melanin-mediated photochemistry, contributing to the generation of “dark CPDs”, a unique class of lesions that persist long after UV exposure has ceased, extending the window of potential DNA damage and mutation accumulation [41].

### 3.1. UVB Radiation in Melanoma Development

UVB radiation plays a pivotal role in melanoma development, with its direct DNA-damaging effects and disruption of repair pathways acting as key carcinogenic mechanisms. In addition to CPD and 6-4PP formation, UVB-induced mutations in *TP53* and *CDKN2A* disrupt critical tumor suppressor pathways, promoting unchecked melanocyte proliferation [42,43,44]. Moreover, *MC1R* signaling, a crucial pathway for both pigmentation and DNA repair, is significantly affected by UVB exposure [41]. When functional, *MC1R* enhances the efficiency of NER by recruiting repair proteins to sites of photodamage. However, in individuals carrying loss-of-function *MC1R* variants, this protective mechanism is weakened, leading to inefficient repair of UVB-induced mutations and an increased susceptibility to melanoma [40].

### 3.2. UVA Radiation in Melanoma Development

While UVB initiates melanoma by directly damaging DNA, UVA, in turn, contributes to melanomagenesis through oxidative stress and mitochondrial dysfunction [45]. The generation of ROS and oxidative DNA lesions, such as 7,8-dihydro-8-oxyguanine (8-oxoG), frequently leads to G>C to T>A transversions, a mutation pattern commonly found in melanoma genomes [46,47,48,49]. Furthermore, UVA exposure promotes the photoisomerization of 6-4PPs into Dewar valence isomers, extending the persistence of DNA damage beyond the initial UV insult [50,51]. Another significant aspect of UVA-induced damage is its impact on mitochondrial integrity. By disrupting mitochondrial function, UVA radiation can trigger the release of pro-apoptotic factors such as cytochrome c, activating intrinsic apoptotic pathways. However, in melanocytes that evade apoptosis, persistent mitochondrial dysfunction fosters genomic instability, a hallmark of tumor initiation and progression [52].

## 4. Infrared Radiation (IRA)/UVR Cross-Talk in Melanoma Development

Beyond the contributions of UVA and UVB, infrared radiation (IR) has also been implicated in modifying UVR-induced skin cancers. Although IR itself is not inherently mutagenic, evidence suggests that infrared A (IRA, 780–1400 nm), the predominant terrestrial IR component, may interfere with apoptosis and enhance the survival of UV-damaged melanocytes [53]. IRA has been shown to reduce UVB-induced apoptosis by modulating the extrinsic apoptotic pathway, inhibiting caspase-8 activation, and altering the expression of apoptosis-related proteins (BID, BAX, Bcl2, and FLIP_L_). While this may protect normal skin cells from excessive UVR-induced damage, it also allows DNA-damaged melanocytes to evade cell death, increasing the likelihood of mutation accumulation and malignant transformation. Notably, IRA does not appear to affect UVB-induced DNA repair, meaning that while damaged cells survive longer, their DNA remains unrepaired, further promoting melanomagenesis [54,55,56]. The influence of IRA on UVR-induced ROS in human melanocytes needs further investigation.

## 5. Genetic Predisposition

While UVR-induced DNA damage is a major driver of melanoma, genetic factors also play a significant role in determining individual susceptibility to the disease. Melanoma arises from a complex interplay between inherited (germline) mutations and acquired (somatic) mutations that are pivotal in predisposing individuals to melanoma (Table 1) [40,57,58,59,60,61,62,63,64,65,66,67,68,69,70,71,72,73,74,75,76,77,78,79,80,81,82,83,84,85,86,87,88,89,90,91,92,93,94,95,96,97,98,99,100,101,102,103]. These mutations affect a variety of cellular components, including genes involved in pigmentation, key tumor suppressor genes and oncogenes, crucial signaling pathways, and other important players, such as transcription factors and epigenetic modifications [104,105,106,107].

Mutations in the *MC1R* gene are among the most well-established genetic risk factors for melanoma. They not only increase UV sensitivity but also impair DNA repair, leading to a twofold increase in melanoma risk. Moreover, *MC1R* mutations have a synergistic effect with other melanoma-associated mutations, particularly in *CDKN2A* and *BRAF*, further amplifying melanoma risk [40,58].

Nonetheless, *TP53*, one of the most frequently mutated genes in cancer, is also commonly affected in melanoma. UVR-induced mutations in *TP53* impair its function as a tumor suppressor, allowing for unchecked cell proliferation and increased survival of UV-damaged melanocytes [108,109]. Additionally, mutations in the *NF1* gene, often observed in melanomas arising on chronically sun-exposed skin [77], lead to hyperactivation of the *NRAS–MAPK* pathway and increased resistance to apoptosis [78].

Another critical pathway involved in melanoma pathogenesis is the *RAS–RAF–MEK–ERK (MAPK)* signaling pathway. Activating mutations in *BRAF*, particularly the V600E mutation, are found in approximately 50% of melanomas. This mutation drives constitutive activation of *MEK* and *ERK*, promoting melanocyte proliferation and survival. While *BRAF* mutation is a frequent initiating event that activates the MAPK pathway, the inactivation of key tumor suppressor genes, such as *CDKN2A*, *PTEN*, and *TP53*, is crucial for overcoming *BRAF*-induced senescence and allowing the uncontrolled proliferation and survival that characterize melanoma. Importantly, UVR exposure leads to impaired *TP53* function, accelerating the progression of *BRAF*-driven melanoma [108,110].

Mutations in *NRAS* and *KIT* similarly enhance *MAPK* signaling, contributing to tumor growth and metastasis [111].

Finally, epigenetic modifications, such as DNA methylation and histone modification, further complicate the genetic landscape of melanoma. Aberrant methylation of tumor suppressor genes and oncogenes has been implicated in melanoma progression, with specific methylation signatures correlating with increased metastatic potential [112].

## 6. Cellular Response to UV-Induced DNA Damage

**Figure 1:**
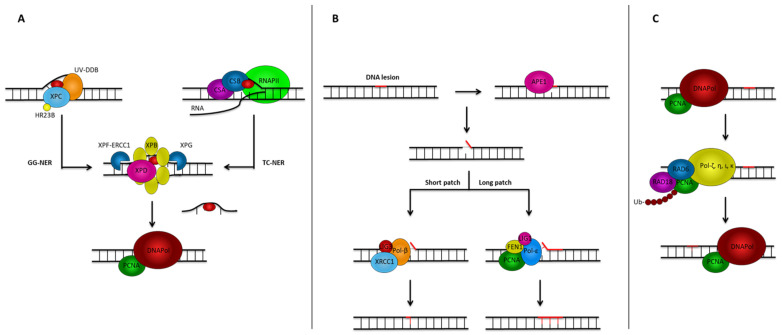
The image illustrates the three main DNA repair mechanisms: (**A**) NER, (**B**) BER, and (**C**) TLS. XPC: Xeroderma Pigmentosum Complementation group C; HR23B: Homologous Recombination 23B; UV-DOB: UV-Damage DNA Binding; XPF-ERCC1: Xeroderma Pigmentosum Complementation group F-Excision Repair Cross-Complementation group 1; XPB: Xeroderma Pigmentosum Complementation group B; XPG: Xeroderma Pigmentosum Complementation group G; DNAPol: DNA Polymerase; PCNA: Proliferating Cell Nuclear Antigen; RNAPII: RNA Polymerase II; CSB: Cockayne Syndrome group B-protein; XPD: Xeroderma Pigmentosum Complementation group D; APE1: Apurinic/Apyrimidinic Endonuclease 1; Pol-β: DNA Polymerase β; XRCC1: X-Ray Repair Cross-Complementing protein 1; FEN1: Flap Endonuclease 1; Pol δ/ε: DNA Polymerases δ and ε; Ub: ubiquitin.

DNA damage can be addressed through two fundamental repair mechanisms: (1) direct damage repair and (2) removal and replacement of damaged DNA regions. While photoreactivation (a FAD-dependent direct repair mechanism) occurs in bacteria [105], human cells rely primarily on excision and replacement strategies (Figure 1). Interestingly, UVR triggers α-MSH release from keratinocytes and melanocytes [106,107,108,109,110,111], thus contributing to the activation of the downstream signaling pathways that modulate NER and BER to enhance genomic stability and resist UV-mediated apoptosis [5,6,7,8,9,10,11,12].

### 6.1. Nucleotide Excision Repair (NER)

NER serves as the primary repair mechanism for UV-induced DNA damage through two sub-pathways: (1) Global Genomic NER (GG-NER), in which the UV-DDB initially recognizes DNA lesions and recruits the XPC-HR23B complex to the site of the lesion; and (2) Transcription-Coupled NER (TC-NER), in which stalled RNA polymerase II recruits repair machinery [112].

The repair process involves unwinding the DNA helix by XPD (ERCC2) and XPB (ERCC3) helicases, excision of the damaged strand by XPF-ERCC1 and XPG endonucleases, followed by DNA synthesis using the undamaged strand as a template and ligation.

NER regulation involves damage sensors (XPC-HR23B, RNAPII/CSB, ATM/ATR) that trigger downstream signaling through p53, CHK1/2, and BRCA1, affecting cell cycle checkpoints and repair [113]. Post-translational modifications (phosphorylation, ubiquitination, acetylation) coordinate repair protein activity. NER integrates with cell cycle checkpoints through ATM/ATR activation of TP53 and CHK1/CHK2, arresting cells at G1/S, while BRCA1/2 proteins facilitate repair during the S and G2/M phases [114,115].

### 6.2. Base Excision Repair (BER)

Although UVR primarily causes pyrimidine dimers (repaired by NER), it can also induce oxidative stress, leading to base lesions repaired by BER. It operates through two sub-pathways: (1) Short-Patch BER, which replaces a single nucleotide using DNA polymerase β and DNA ligase III/XRCC1; and (2) Long-Patch BER, which synthesizes 2–10 nucleotides using polymerases δ/ε, PCNA, FEN1, and DNA ligase I [116,117,118]. BER is tightly regulated through DNA damage sensors, repair proteins, and post-translational modifications [119].

This process involves (1) DNA glycosylases removing damaged bases to generate AP sites; (2) APE1 creating single-strand breaks at AP sites [120]; (3) DNA polymerase β filling the nucleotide gap [121]; and (4) DNA ligase sealing the strand break.

Also, for oxidative damage like 8-oxoG, which pairs with adenine and causes mutations, MUTYH removes incorrect adenines while OGG1 excises 8-oxoG paired with cytosine [1]. PNKP processes damaged DNA ends through 3′-phosphatase and 5′-kinase activities to generate suitable ends for repair [122].

### 6.3. Translesion Synthesis (TLS)

TLS is a DNA damage tolerance mechanism that allows replication to bypass lesions (like UV-induced thymine dimers or AP sites) that would otherwise stall replication, leading to genomic instability, cell death, or mutations. It uses specialized, error-prone TLS polymerases to allow replication to proceed despite the presence of lesions [123]. When replicative polymerase encounters a lesion, PCNA is ubiquitinated by RAD6/RAD18, triggering polymerase switching to specialized TLS polymerases. Several TLS polymerases exist, each with specific properties. For instance, Pol ζ bypasses 6-4 photoproducts with high fidelity [124], while Pol η efficiently bypasses CPDs, though deaminated cytosine residues in CPDs lead to C→T signature mutations [125].

The incorporation of incorrect nucleotide bases across from UVR-induced DNA lesions by error-prone DNA polymerases during TLS is mutagenic. The mismatch repair proteins Msh2/Msh6 recognize incorrect nucleotides incorporated during TLS and trigger their removal, creating single-stranded DNA patches that must be filled before replication to prevent double-strand breaks and apoptosis [126]. Moreover, Msh2/Msh6 deficiency increases mutation frequency.

## 7. UVR-Mediated Inflammation and Immunosuppression in Skin Carcinogenesis

Chronic UVR exposure causes skin aging and significantly increases the risk of skin cancer. UVR-absorbing chromophores initiate a series of biochemical and immunologic events that lead to UVR-induced injury. In the initial phase, keratinocytes undergo apoptosis in large numbers primarily by the activation TP53 [127] and the death receptor CD95/Fas [128]. These apoptotic keratinocytes release pro-inflammatory cytokines into the skin microenvironment, including TNF-α, IL-1α, IL-1β, GM-CSF, IL-6, IL-8, and IL-10 [129]. Along with these, alarmins also contribute to inflammation. Alarmins are intracellular molecules that act as pro-inflammatory mediators in the extracellular milieu upon damage. When released, alarmins bind pattern recognition receptors (PRRs) or other surface receptors, resulting in NF-κB activation and subsequent pro-inflammatory cytokine production. Indeed, evidence has highlighted that UV radiation stimulates HMGB1 release in keratinocytes in vitro and HMGB1 is expressed in skin tumors after chronic radiation [130,131]. These cytokines play a crucial role in orchestrating the inflammatory response and subsequent tissue repair. The recruitment of macrophages via the CCR2/CCL2 axis (chemokine receptor CCR2 and its ligand CCL2) is a critical step in establishing a pro-tumorigenic microenvironment [132].

In addition to its inflammatory effects, UVR is a potent immunosuppressant in the skin. Immunosuppression begins locally in the irradiated area but can have systemic effects [133]. Both UVA and UVB radiation impact the immune system, although most studies on UVR-induced immunosuppression have focused on UVB [134,135]. Under normal circumstances, the transformed cells are recognized and eliminated by anti-tumor immune responses elicited by the host. However, the strong immunosuppressive effects of UVR can allow these transformed cells to evade immune surveillance [133,136,137]. Furthermore, during melanoma progression, melanoma cells are able to avoid immune response via the programmed cell death-1/programmed cell death ligand-1 (PD-1/PD-L1) pathway. Activated T cells and tumor-infiltrating lymphocytes are blocked by the binding of PD-1 with PD-L1 induced by tumor cells, which act as a negative factor for immunomodulation [138].

## 8. Pathogenesis of Melanoma: Diagnostic and Surgical Controversies

### 8.1. Diagnostic Aspects

In the diagnostic context of melanoma, factors related to UV exposure and skin phenotype, as well as genetic factors, are critical to establishing screening schedules for early melanoma detection [139]. Considering the aggressive nature of invasive melanoma, timely diagnosis significantly improves patient outcomes. Risk assessment should include sun exposure history, particularly childhood sunburns and adult tanning habits; phenotypic traits, as fair skin, red or blonde hair, and light eyes indicate increased susceptibility; and high nevus count and genetic factors, as, for instance, *MC1R* gene variants, associated with red hair phenotype, confer increased melanoma risk due to reduced protective pigmentation [33]. Multivariate risk models that integrate these factors enhance stratification accuracy, though reliance on self-reported exposure may introduce bias [140,141].

In this context, dysplastic nevi—while not direct melanoma precursors—are considered important clinical markers of elevated melanoma risk. They may display some architectural disorder and cytologic atypia, but most do not progress to melanoma. Their presence, especially in patients with multiple lesions or a family history of melanoma, supports intensified surveillance strategies. UVR exposure in these individuals contributes to cumulative mutational burden rather than a linear dysplastic nevus–melanoma progression pathway.

Advanced non-invasive imaging techniques, such as dermoscopy and reflectance confocal microscopy (RCM), have transformed non-invasive melanoma diagnosis. They have improved early melanoma detection rates, particularly for atypical presentations on photodamaged skin through widely consolidated assessment algorithms [142,143,144,145]. RCM offers cellular-level visualization, and it increases sensitivity and confidence in diagnosis, as proven by a recent randomized controlled trial [146]. However, diagnosis can be challenging due to skin variations associated with skin photodamage and potential overlapping features between benign and malignant lesions. Nevertheless, histopathological analysis represents the gold standard for diagnosing melanocytic lesions, although distinguishing lesions arising on photoexposed skin remains challenging [147,148].

Currently, artificial intelligence (AI) applications in melanoma detection have shown promising results, with convolutional neural networks demonstrating diagnostic accuracy comparable or superior to dermatologists in controlled studies [149]. These systems analyze dermoscopic images using deep learning algorithms trained on extensive datasets. Total-body photography combined with AI-assisted sequential digital dermoscopy enables comprehensive monitoring of high-risk patients, facilitating detection of subtle changes indicative of early melanoma. Mobile applications leveraging these technologies may expand access to preliminary melanoma screening, though concerns regarding algorithmic bias, data privacy, and clinical integration remain significant challenges.

### 8.2. Molecular and Histopathologic Characteristics and Their Implications in Clinical Practice

#### 8.2.1. Molecular Biomarkers in Clinical Practice

Recent advances in molecular diagnostics have led to the integration of several key genetic and circulating biomarkers in melanoma care.

These include *BRAF*, *NRAS*, and *NF1* mutations, which provide insight into tumor biology and guide therapeutic decisions. Liquid biopsy tools—such as circulating tumor DNA (ctDNA), microRNAs (miRs), and exosomes—allow for non-invasive monitoring. ctDNA levels correlate with tumor burden and treatment response, while miR signatures (e.g., miR-15b, miR-150, miR-425) are under investigation for early detection and prognostication.

#### 8.2.2. Immunohistochemical Biomarkers

Immunohistochemistry (IHC) remains a cornerstone in melanoma diagnosis and classification. Markers such as PD-L1 help stratify patients for immune checkpoint therapies. Additionally, S100B and LDH serve as serum markers for disease monitoring in advanced stages, although their sensitivity in early-stage melanoma is limited. These molecular and IHC biomarkers not only aid in diagnosis but also reflect the tumor’s biological behavior and immune interactions.

#### 8.2.3. Etiopathogenic and Molecular Divergence by Sun Exposure Pattern

These biomarker profiles also mirror the underlying etiopathogenic differences between melanomas arising on chronically sun-damaged (CSD) versus non-sun-damaged (non-CSD) skin. CSD melanomas exhibit higher mutational burdens dominated by UV signature mutations (C→T transitions), frequent activating mutations in *NRAS* and *NF1* and the inactivation of tumor suppressors such as *CDKN2A* and *TP53.* These tumors typically develop through a gradual progression model from precursor lesions. In contrast, non-CSD melanomas show lower mutational burden, prevalent BRAFV600E mutations, and less genomic complexity, often arising without identifiable precursor lesions. These molecular distinctions have significant implications for prognosis and treatment response, with CSD melanomas generally demonstrating greater heterogeneity and potential for immune recognition [150,151,152,153,154,155,156,157,158,159].

This molecular divergence aligns with the recent World Health Organization (WHO) histopathological classification, which categorizes melanoma into high and low CSD subtypes. High CSD melanoma includes lentigo maligna and desmoplastic melanoma, while low CSD melanoma primarily includes superficial spreading melanoma, further highlighting distinct etiopathogenic and genomic profiles [160,161].

For instance, tumor mutational burden (TMB) and immunotherapy response in melanoma represent a critical aspect of precision oncology. UV-induced melanomas typically exhibit high TMB, generating numerous neoantigens that serve as targets for immune recognition. Clinical evidence demonstrates superior responses to immune checkpoint inhibitors (anti-PD-1/PD-L1, anti-CTLA-4) in melanomas with higher TMB, particularly those with UV signature mutations. This correlation provides a mechanistic explanation for the paradoxical observation that melanomas with indicators of greater sun damage often show improved survival outcomes with immunotherapy [162]. However, response patterns remain complex, with factors beyond TMB, including tumor microenvironment composition and immune exclusion mechanisms, significantly modulating treatment efficacy. Ongoing research aims to develop predictive biomarkers that integrate mutational signatures with immune infiltration patterns to optimize immunotherapeutic approaches.

### 8.3. Surgical Management

Furthermore, surgical management of melanoma in photodamaged skin requires tailored approaches, particularly in chronically sun-exposed regions such as the face, upper limbs, and shoulders, due to cosmetic and functional reasons [163]. Accurate assessment of lesion depth, evaluation of margins, and surveillance after excision are essential in managing melanoma, especially those arising in chronically sun-damaged skin [164].

### 8.4. Controversies

Despite significant progress in understanding the link between melanoma and photodamage, controversies persist, particularly regarding the role of sun exposure in melanoma pathogenesis. While intermittent, high-intensity UV exposure—especially in childhood—clearly elevates melanoma risk, chronic sun exposure in outdoor workers has shown inconsistent associations with melanoma development. Paradoxically, melanoma incidence has increased more among indoor workers, leading researchers to explore other contributing factors like genetic predisposition and artificial tanning [165]. Another point of debate concerns the fact that, despite the increase in solar exposure over the years, only a small portion of new melanoma diagnoses are classified as LM, the subtype most closely associated with sun exposure. This can be explained from various points of view. LM typically develops over many years due to chronic sun exposure. Consequently, even with increased exposure, it may take a long time for it to manifest. This means that the current diagnosis rates may not fully reflect the recent increases in UV exposure.

However, it is important to note that primary prevention efforts targeting UV exposure have shown mixed results in modulating melanoma incidence trends. Educational campaigns promoting sun-protective behaviors have increased public awareness but demonstrated limited impact on behavior modification. Structural interventions including shade provision in public spaces and UV index warnings have shown greater effectiveness. Also, school-based programs targeting children have demonstrated promising results in establishing early sun-protective habits, while workplace policies for outdoor workers remain inconsistently implemented. Notably, Australia’s comprehensive “SunSmart” program, combining education, environmental changes, and policy development, has achieved stabilization of melanoma rates among younger cohorts, providing evidence that long-term, multi-level interventions can effectively reduce melanoma incidence [166].

Finally, other factors, such as genetic mutations and environmental factors, may contribute to the prevalence of different melanoma subtypes, leading to a more significant number of cases that are not classified as LM. Finally, while overall UV exposure may have increased, changes in lifestyle and sun protection behaviors can impact the incidence of specific melanoma subtypes. For instance, using sunscreen, protective clothing, and shade-seeking behaviors may contribute to a lower incidence of LM, even in those with considerable sun exposure. In summary, while increased solar exposure contributes to the risk of melanoma, the relationship between UV exposure and specific melanoma subtypes is complex and influenced by various biological, behavioral, and environmental factors.

### 8.5. Future Directions and Clinical Perspectives

Despite ongoing debate around the precise role of chronic sun exposure in melanoma pathogenesis, future research should aim to delineate the molecular events occurring during the earliest phases of UV-induced photodamage. Markers of oxidative DNA damage, such as 8-oxoG and persistent CPDs, have been proposed as indicators of photogenotoxic stress and mutational load in pre-neoplastic skin [167]. Additionally, pro-inflammatory alarmins (e.g., HMGB1) and cytokines such as IL-1β, TNF-α, and IL-6 are increasingly recognized as molecular mediators of the tumor-promoting microenvironment following chronic UV exposure [13,130]. Alterations in immunosenescence-related pathways and Langerhans cell depletion have also been implicated in the failure of immune surveillance in photoexposed skin [168].

Clinically, the integration of liquid biopsy technologies—including ctDNA, UV-induced miRNAs, and exosomal RNA profiles—with non-invasive diagnostic imaging and AI-supported skin surveillance tools could enable earlier melanoma detection in high-risk individuals [169]. Moving forward, the development of integrated biomarker panels, combining genomic, epigenomic, inflammatory, and immune features, may enable a personalized prevention and early detection model, particularly in populations with high cumulative sun damage.

## 9. Conclusions

UVR plays a pivotal role in melanomagenesis by inducing DNA damage, promoting oxidative stress, and creating a pro-inflammatory and immunosuppressive skin microenvironment. Despite the presence of DNA repair systems such as NER and BER, chronic UVR exposure often overwhelms these defenses, contributing to mutational burden, immune evasion, and the transformation of melanocytes. Recent insights into the molecular and histopathologic divergence between melanomas arising on CSD versus non-sun-damaged skin have revealed important genomic and immunologic differences, with significant implications for prognosis and therapeutic responsiveness—particularly to immunotherapies.

Advanced molecular and immunohistochemical biomarkers, including ctDNA, miRNAs, *BRAF/NRAS/NF1* mutations, and PD-L1 expression, are increasingly integrated into diagnostic and management pathways. These tools, together with non-invasive imaging and AI-supported surveillance, pave the way for earlier detection and personalized prevention strategies.

Controversies persist regarding the role of chronic sun exposure and melanoma subtype prevalence, but ongoing research into photodamage-associated biomarkers and UVR-driven molecular signatures holds promise for resolving these uncertainties. Looking ahead, the development of integrated biomarker panels, capable of detecting early photoinduced molecular alterations, will be critical to improving outcomes. A refined understanding of the intersection between UVR, genetic susceptibility, immune modulation, and diagnostic technologies is essential to advancing melanoma prevention, risk stratification, and treatment.

## Figures and Tables

**Table 1:** The table describes the main genetic factors that predispose to the development of melanoma.

Category	Gene/Pathway	Mutation Type	Effect	Melanoma Risk	Reference
Pigmentation	*MC1R*	Variants (especially red hair color—RHC-associated)	Alters pigmentation, UV sensitivity, DNA repair	Increased (doubles risk with *CDKN2A* mutations)	[33,50,51]
	*ASIP*	Variants	Alters melanocortin signaling	Slightly increased	[50]
	*TYR*	Variants	Alters melanin synthesis	Slightly increased	[50]
Tumor Suppressor	*CDKN2A* (p16/INK4a, p14/ARF)	Germline mutations, Loss of function	Deregulates cell cycle, impairs senescence, affects oxidative stress	High (67% lifetime risk with heterozygous loss)	[52,53,54,55,56,57,58,59,60,61,62,63,64]
	*RB*	Germline mutations	Reduces tumor suppressor activity	Increased	[65,66]
	*PTEN*	Inactivating mutations, deletions, epigenetic silencing	Activates AKT signaling	Increased	[67,68]
	*NF1*	Loss-of-function mutations	Hyperactivates NRAS, MAPK, and PI3K/AKT pathways	Increased (especially with sun damage)	[69,70]
Signaling Pathway (MAPK)	*BRAF*	V600E (most common), V600R, V600D	Constitutive activation of kinase activity, activates MEK/ERK	Highly increased	[71,72,73,74,75,76]
	*NRAS*	Q61R, Q61K (most common)	Activates MAPK pathway	Increased	[77,78]
	*RAS (KRAS, HRAS, NRAS)*	Mutations	Activates RAF kinases	Increased	[76]
Signaling Pathway (WNT)	*CTNNB1*	Mutations	Stabilizes β-catenin, activates transcription	Increased (2–23% of cases)	[79,80]
Receptor Tyrosine Kinase (RTK)	*EGFR*	Gene copy number gains, point mutations	Dysregulation	Increased	[81]
	*HGF/MET*	Gene copy number gains, point mutations	Dysregulation	Increased	[82]
	*KIT*	L576P mutation	Activates KIT signaling	Increased (small number of melanomas)	[83]
	*PTPRD*	Deletions	Loss of phosphatase activity	Increased	[84]
	*PDGFR, IGFR*	Upregulation	Increased signaling	Increased	[85]
Transcription Factor	*MITF*	Amplification, E318K mutation	Drives melanocytic lineage, survival, growth, differentiation	Increased	[86,87,88,89]
	*MYC*	Overexpression	Enhances melanoma progression	Increased	[90]
	*TBX2*	Amplification	Represses p14ARF and p21CIP1	Increased	[91,92]
	*TERT*	Promoter mutations (C→T)	Creates ETS transcription factor binding sites	Increased proliferation	[93,94]
Epigenetic Factors	Multiple genes	Differential methylation (UV exposure signature)	May drive melanoma development	Increased	[95,96]

## Abbreviations

The following abbreviations are used in this manuscript:

6-4PPs	6-4 photoproducts
8-oxodGuo	7,8-dihydro-8-oxyguanine
AI	Artificial Intelligence
BER	Base Excision Repair
CDPs	Cyclobutane Pyrimidine Dimers
CM	Cutaneous Melanoma
CSD	Chronically Sun-Damaged
ctDNA	Circulating Tumor DNA
iNOS	Inducible Nitric Oxide Synthase
IR	Infrared Radiation
LM	Lentigo Maligna
LMM	Lentigo Maligna Melanoma
NO	Nitric Oxide
NOX	NADPH Oxidase
NER	Nucleotide Excision Repair
O_2_^−^	Superoxide Radical Ions
O_3_	Ozone
ONOO^−^	Peroxynitrite
RCM	Reflectance Confocal Microscopy
ROS	Reactive Oxygen Species
TLS	Translesion Synthesis
TMB	Tumoral Mutation Burden
UVR	Ultraviolet Radiation
